# Coronary Lesions and Systemic Inflammatory Response Syndrome in Kawasaki Disease

**DOI:** 10.31662/jmaj.2018-0013

**Published:** 2018-12-17

**Authors:** Yutaro Tomobe, Osamu Nomura, Yoshihiko Morikawa, Nobuaki Inoue, Hiroshi Sakakibara, Masaru Miura

**Affiliations:** 1 Department of General Pediatrics, Tokyo Metropolitan Children’s Medical Center, Tokyo, Japan; 2 Division of Pediatric Emergency Medicine, Tokyo Metropolitan Children’s Medical Center, Tokyo, Japan; 3 Clinical Research Support Center, Tokyo Metropolitan Children’s Medical Center, Tokyo, Japan; 4 Department of Cardiology, Tokyo Metropolitan Children’s Medical Center, Tokyo, Japan

**Keywords:** Kawasaki disease, Kobayashi score, systemic inflammatory response syndrome, vital signs, coronary artery lesion

## Abstract

**Introduction:**

In patients with Kawasaki disease (KD), who later develop coronary artery lesions (CALs), several inflammatory cytokines are reportedly higher than in patients without CALs. Systemic inflammatory response syndrome (SIRS) is used as a clinical index of hypercytokinemia. The objective of this study was to determine whether SIRS is related to CAL formation.

**Methods:**

We conducted a retrospective cohort study of KD patients admitted to our hospital between July 2012 and July 2015. The subjects were classified into the SIRS or the non-SIRS group based on their vital signs and blood test results. Their initial treatment was determined by their Kobayashi score. We compared the incidence of CALs between the two groups.

**Results:**

Of 357 KD patients, 277 were included in this study and 175 (63.2%) met the SIRS criteria. The incidence of CAL formation at week 1 in the clinical course and at one month after the primary treatment was significantly higher in the SIRS group than in the non-SIRS group (17.7% vs. 7.8%, *p* = 0.03 and 10.9% vs. 3.9%, *p* = 0.03, respectively). Multivariate analyses showed that after adjusting for each variable of the Kobayashi score, SIRS was an independent risk factor for CAL formation at week 1 in the clinical course (odds ratio, 2.7; 95% confidence interval, 1.03–7.23; *p* = 0.04).

**Conclusions:**

SIRS can be a risk factor for CAL in the acute phase of KD.

## Introduction

Kawasaki disease (KD) is an acute, self-limiting vasculitis of childhood characterized by coronary artery lesion (CAL) development, its main complication. KD is currently the most common cause of pediatric acquired heart disease in developed countries ^(1)^. The main pathophysiology of KD is the elevation of inflammatory cytokines inducing migration of inflammatory cells into the coronary arteries, as well as the dissociation and destruction of the internal elastic lamina resulting in the formation of CALs ^(2), (3)^.

Systemic inflammatory response syndrome (SIRS) ^(4)^ was introduced in 1992 by the American College of Chest Physicians and the Society of Critical Care Medicine as a clinical index of the patient’s response to both infectious and non-infectious insults including severe bacterial infection, trauma, and burn injury. SIRS is defined as the presence of two or more of the following variables: body temperature (BT) exceeding 38.0℃, heart rate (HR) exceeding 90 beats per minute, respiratory rate (RR) exceeding 20 breaths per minute, and an abnormal white blood cell (WBC) count, with one of the two necessary variables being either BT or WBC. These physiological changes are thought to result from the excessive elevation of cytokines ^(5), (6)^. In 2005, a consensus was reached on the definition of pediatric SIRS ^(7)^. According to this definition, pediatric SIRS must include two or more of the following, one of which must be an abnormal temperature (a core BT of < 36℃ or >38.5℃) or abnormal leukocyte count, tachycardia (or bradycardia in infants), tachypnea, elevated or depressed WBC count for the patient’s age, or >10% immature neutrophils.

We hypothesized that KD patients meeting the SIRS criteria would have higher levels of inflammatory cytokines and a higher attendant risk of CAL formation. However, the relationship of KD with SIRS in CAL formation has not been established. The objective of this study was to compare the incidence of CAL in patients with or without SIRS at the time of KD diagnosis and to determine whether SIRS is a risk factor for CAL formation.

## Materials and Methods

### Study design and patients

We performed a retrospective cohort study by reviewing patient records. Data used for this study were obtained from KD patients seen between July 2012 and July 2015 at the Tokyo Metropolitan Children’s Medical Center in Tokyo, Japan. The vital signs of each patient, including BT (in the axilla), HR, and RR, were recorded by nurses trained in emergency department triage (ED) when the patients first arrived at our hospital and before they received any medical interventions. The vital signs obtained at triage were used as the basis for this study irrespective of the patients’ emotional state and behavior such as crying or sleeping. This study included patients who received the diagnosis of Kawasaki disease in the emergency department in accordance with the Japanese diagnostic criteria, including those with a diagnosis of incomplete Kawasaki disease ^(8)^. Subjects were excluded if they had incomplete documentation of vital signs and echocardiogram results, were observed for less than four weeks, were afebrile on admission, or had a past history of KD.

### Clinical outcomes

The participants were classified into the SIRS or non-SIRS group based on their vital signs (BT, HR, and RR) and blood test results (WBC) in accordance with the SIRS criteria ^(7)^ of the 2005 International Pediatric Sepsis Consensus Conference. These data were obtained on the day of the patients’ visit to our ED before their admission for KD. The clinical course of the two groups was compared one month after treatment. The primary outcome was the incidence of CAL at week 1 of the initial treatment. The secondary outcome was the incidence of resistance to initial treatment as described below. We defined “resistance to initial treatment” as a clinical course in which patients with a persistent or recurrent fever lasting more than 24 hours after completion of the primary treatment received additional intravenous immunoglobulin (IVIG) as a second-line rescue treatment. “Afebrile” was also defined as an axillary temperature of < 37.5℃ for more than 24 hours.

Echocardiograms for cardiac function and coronary artery status were performed by ultrasonographers according to the standardized method ^(9)^, and certificated pediatric cardiologists reviewed the ultrasonography images and video recordings. A coronary artery lesion was defined in accordance with the 2004 statement of the American Heart Association ^(10)^ as a Z score of ≥2.5 for the left anterior descending coronary artery or right coronary artery, or alternatively as coronary artery conditions meeting the Japanese Ministry of Health criteria for an aneurysm ^(11)^ (maximum diameter of ≥3.0 mm in children aged < 5 years and ≥4.0 mm in children aged ≥5 years, a diameter of ≥1.5 times that of an adjacent segment, or a clearly irregular luminal contour). Echocardiogram data were obtained serially before treatment and at week 1 (days 5 to 9) and month 1 (days 20 to 50) after treatment.

### Outline of the treatment and stratification by risk score

Every KD patient was assigned a Kobayashi score, a risk score ^(12), (13)^ predicting responsiveness to initial IVIG treatment. The scoring and cut off values were as follows: two points each for a serum sodium concentration of ≤ 133 mmol/L, ≤ 4 days of KD at diagnosis, aspartate aminotransferase (AST) concentration of ≥100 U/L, and WBC count consisting of ≥80% neutrophils; and one point each for a platelet count ≤ 30×10^4^/μL, C-reactive protein (CRP) concentration of ≥10 mg/dL, and age of ≤ 12 months. The patients were classified into two groups by their risk score. As initial treatment, the low-risk group (score ≤ 4) received IVIG (2 g/kg/day for 1 day) while the high-risk group (score ≥5) received IVIG plus prednisolone (2mg/kg/day in three divided doses). The patients also received aspirin (30 mg/kg/day), the dosage of which was reduced to 5 mg/kg/day a few days after the abatement of fever.

### Statistical analysis

Data were presented as the mean ± standard deviation for continuous variables or as a percentage for categorical variables. Categorical data were compared between the two groups using the Fisher’s exact test, and continuous data were analyzed using the two-sample *t*-test. Multiple logistic regression analysis was performed, and the odds ratios (ORs) were adjusted for the risk score variables (age, number of days of illness, days of initial treatment, percentage of neutrophils, platelet count, AST, and CRP). For all analyses, a two-sided *p* < 0.05 was considered statistically significant. All analyses were performed using SPSS version 23.0 (IBM Corp. Armonk, NY, USA).

This study was approved by the ethics board of the Tokyo Metropolitan Children’s Medical Center (ID: H25-24). The study protocol conformed to the ethical guidelines of the Declaration of Helsinki and those of the Ministry of Education, Culture, Sports, Science and Technology and the Ministry of Health, Labour and Welfare of Japan for medical and health research involving human subjects. Due to the retrospective nature of the study (data were collected from patient charts), informed consent was not obtained in accordance with the above guidelines.

## Results

**Figure 1.**
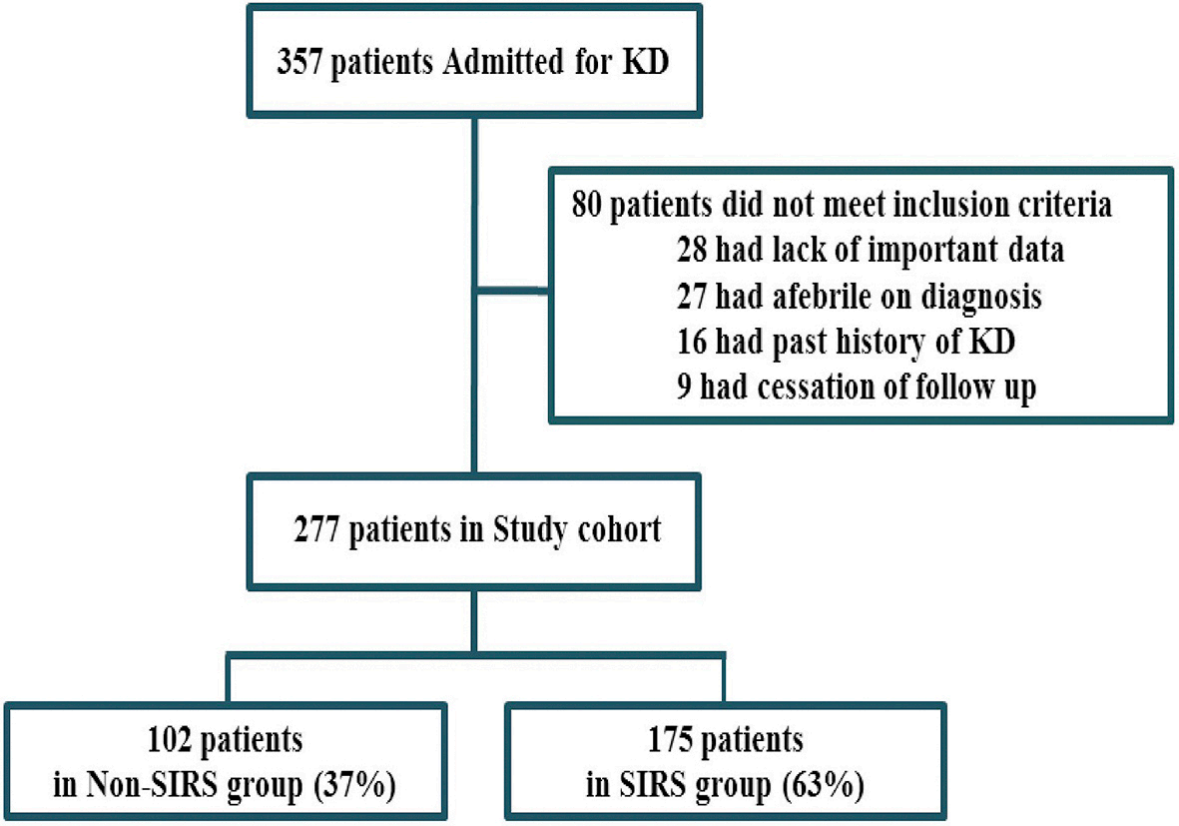
Derivation of the study population. All subjects were classified into the SIRS or non-SIRS group according to their vital signs and blood test results on the day of admission. KD: Kawasaki Disease, SIRS: Systemic Inflammatory Response Syndrome.

Among 357 KD patients examined during the study period (Figure 1), 80 were excluded because they met the exclusion criteria. Of the 277 patients who were included, 175 (63%) met the SIRS criteria. Table 1 shows the baseline characteristics and laboratory findings of the two groups. Twenty-one patients in the non-SIRS group and 63 patients in the SIRS group with a risk score of ≥5 received IVIG plus prednisolone. The SIRS group had a higher proportion of older children presenting a higher BT, HR, and WBC. In the laboratory tests, patients with SIRS showed a significantly higher percentage of neutrophils, transaminase (AST and alanine aminotransferase), and CRP, and lower sodium, and thus a higher mean risk score.

**Table 1.** Demographic Data and Laboratory Characteristics of the Two Groups.

	non-SIRS	SIRS	*p*-value
	N = 102	N = 175	
Gender (male)	62 (60.8)	102 (58.3)	0.71
Age (months)	25.5 (15.7)	34.6 (23.9)	< 0.001
Days of illness	4.12 (1.86)	4.20 (2.18)	0.77
Vital signs			
Body temperature (℃)	38.3 (0.51; 102)	39.1 (0.70; 175)	< 0.001
Heart rate (bpm)	152 (22.1; 101)	159 (25.0; 175)	0.02
Respiratory rate (/min)	33.4 (7.83; 87)	34.6 (9.62; 157)	0.29
Laboratory findings			
WBC (10^3^/µL)	12.2 (3.52; 101)	14.9 (4.82; 174)	< 0.001
Neutrophils (%)	62.6 (15.5; 101)	71.1 (15.4; 174)	< 0.001
Platelets (10^4^/µL)	34.9 (12.7; 100)	34.3 (12.4; 172)	0.77
Sodium (mmol/L)	135 (2.89; 101)	133 (2.94; 172)	0.001
AST (U/L)	64.6 (83.2; 101)	113 (242; 172)	0.02
ALT (U/L)	70.7 (113; 101)	108 (165; 174)	0.03
CRP (mg/dL)	6.29 (4.45; 101)	7.50 (4.03; 174)	0.02
Albumin (g/dL)	3.66 (0.47; 99)	3.57 (0.40; 170)	0.09
Total cholesterol (mg/dL)	148 (24.0; 94)	144 (27.2; 157)	0.29
Kobayashi score	2.93 (2.53; 102)	3.81 (2.63; 175)	0.01
Kobayashi score ≥ 5	21 (20.6)	63 (36.0)	0.007

Data are expressed as n (%) or mean (SD; n). WBC, white blood cell count; AST, aspartate aminotransferase; ALT, alanine aminotransferase; and CRP, C-reactive protein

The clinical outcomes of the acute phase after the initial treatment are shown in Table 2. The incidence of CAL at week 1 of the clinical course was significantly higher in the SIRS group than in the non-SIRS group (17.7% vs. 7.8%, *p* = 0.03). The incidence of CAL at 1 month after the primary treatment was also higher (10.9% vs. 3.9%, *p* = 0.03). The difference in the Z scores of each coronary artery branch was not significant. The incidence of resistance to initial treatment did not differ significantly between the two groups (7.0% vs. 10.5%, *p* = 0.36).

**Table 2.** Clinical Outcomes of the Two Groups.

	non-SIRS	SIRS	
	N = 102	N = 175	*p*-value
Z-score of the coronary artery at 1 week of the clinical course			
RCA	1.36 (1.79)	1.54 (1.74)	0.41
LMCA	1.96 (0.83)	2.0 (1.07)	0.74
LAD	1.25 (1.13)	1.42 (1.45)	0.31

Z-score of the coronary artery at 1 month after treatment			
RCA	0.92 (1.84)	1.09 (1.73)	0.45
LMCA	1.63 (0.92)	1.54 (1.0)	0.45
LAD	0.92 (1.16)	0.98 (1.48)	0.71

CAL at week 1	8 (7.8)	31 (17.7)	0.03

CAL at month 1	4 (3.9)	19 (10.9)	0.03
IVIG resistance^a^	10 (10.5)	12 (7.0)	0.36

Data are expressed as n (%) or the mean (SD). The *p*-values were calculated using the Fisher's exact test. RCA, right coronary artery; LMCA, left main coronary artery; LAD, left anterior descending artery; CAL, coronary artery lesion; and IVIG, intravenous immune globulin ^a^ Data on IVIG resistance were unavailable for seven patients with non-SIRS and three patients with SIRS.

The result of the multivariate analysis is shown in Table 3. We determined the OR for SIRS after adjusting for each risk score variable because the initial treatment was expected to influence CAL formation. SIRS was an independent risk factor for CAL formation at week 1 of the KD course (OR, 2.7; 95% confidence interval [CI], 1.03–7.23; *p* = 0.04). On the other hand, the incidence of CAL at 1 month after the primary treatment did not significantly increase despite SIRS (OR, 3.0; 95% CI, 0.81–11.1; *p* = 0.10).

**Table 3.** Multivariate Analyses for CAL at Week 1 and at 1 Month after Treatment.

	CAL at week 1			CAL at month 1		
	OR	95% CI	*p*-value	OR	95% CI	*p*-value
SIRS	2.7	1.03–7.23	0.04	3.0	0.81–11.1	0.10
Kobayashi score variables						
Illness days	1.2	0.96–1.39	0.13	1.2	0.98–1.47	0.08
CRP	1.1	1.03–1.24	0.01	1.1	0.97–1.22	0.14
Neutrophils	1.0	0.98–1.07	0.29	1.0	0.97–1.09	0.31
Platelets	1.0	0.97–1.04	0.83	1.0	0.95–1.05	0.98
AST	1.0	0.99–1.00	0.99	1.0	0.99–1.00	0.65
Sodium	1.0	0.86–1.16	0.99	0.94	0.79–1.13	0.52
Months of age	1.0	0.98–1.002	0.96	1.0	0.97–1.02	0.81

Logistic regression was performed after adjusting for the following Kobayashi score variables: age, days of illness, neutrophil count, platelet count, serum sodium, AST, and CRP. CAL, coronary artery lesion; SIRS, systemic inflammatory response syndrome; OR, odds ratio; CI, confidence interval; CRP, C-reactive protein; and AST, aspartate transferase

## Discussion

These results demonstrated that SIRS was associated with the incidence of CAL in KD patients during the acute phase of the clinical course and was an independent risk factor after adjusting for the risk score variables. On the other hand, resistance to the initial treatment was not significantly related to SIRS. The RAISE study ^(14)^ demonstrated that prednisolone combined with IVIG significantly decreased the unresponsiveness to the initial treatment. Our practical protocol and that of RAISE were based on the Kobayashi score. Because of their higher Kobayashi score, the SIRS group comprised a larger number of the patients who received IVIG plus prednisolone ^(14), (15)^; consequently, neither the incidence of CAL after 1 month nor resistance to initial treatment differed significantly between the SIRS and non-SIRS groups in multivariate analysis.

To the best of our knowledge, this is the first report discussing the relationship of CAL to SIRS in KD. If SIRS predicts the risk of CAL in the acute phase of KD, it can be clinically useful as a rapid indicator of disease severity. Because SIRS assessment is based on the vital signs and WBC count, it can simplify risk assessment and facilitate early identification of changes in the disease. Upon further analysis (Table 4), we found that SIRS effectively predicted the incidence of CAL at week 1 in patients assessed as “low risk” according to their Kobayashi score (i.e., a score of < 5). Virtually, researchers have argued that CAL occurs in such “low risk” patients as well ^(16)^. Given this uncertainty, the SIRS criteria can serve as a ‘safety net’ for identifying patients who might still have a risk of CAL from among those with a Kobayashi score of < 5.

**Table 4.** Clinical Characteristics of the Two Patient Groups with a Kobayashi Score < 5.

	non-SIRS	SIRS	*p*-value
	N = 90	N = 114	
Gender (male)	57 (63.3)	67 (58.8)	0.51
Age (months)	24.6 (18.0)	31.8 (23.4)	0.01
Z-score of the coronary artery at 1 week of the clinical course			
RCA	1.18 (1.19)	1.32 (1.29)	0.44
LMCA	1.98 (0.64)	1.89 (1.01)	0.51
LAD	1.17 (0.94)	1.24 (1.0)	0.57
Z-score of the coronary artery at 1 month after treatment			
RCA	0.80 (1.20)	0.86 (1.21)	0.71
LMCA	1.64 (0.71)	1.44 (0.93)	0.10
LAD	0.91 (0.89)	0.82 (1.03)	0.52
CAL at week 1	5 (5.6)	18 (15.8)	0.03
CAL at month 1	2 (2.2)	9 (7.9)	0.12
IVIG resistance	14 (15.6)	16 (14.0)	0.84

Data are expressed as n (%) or the mean (SD). The *p*-values were calculated using the Fisher's exact test. RCA, right coronary artery; LMCA, left main coronary artery; LAD, left anterior descending artery; CAL, coronary artery lesion; IVIG, intravenous immune globulin ^a^ Data on IVIG resistance were unavailable for five patients with non-SIRS and two patients with SIRS.

In a systematic review ^(17)^, early and aggressive treatment for high-risk KD patients was found to be beneficial in preventing coronary artery complications. The SIRS criteria, which enable early intervention for high-risk patients, have the potential to improve the short-term outcome of coronary artery lesions. As the published risk scores are currently insufficient to predict CAL formation in other cohorts besides those studied in Japan ^(18), (19)^, the SIRS criteria can be used as a predictor of CAL formation to complement the risk scores. KD patients with SIRS may need to be managed carefully as high-risk patients even if they do not have a higher score.

SIRS reflects the physiological changes which result from systemic inflammation and commonly persists if inadequately treated. It indicates the early stages of a disease which may culminate in shock and multiple organ failure ^(20)^. The pathophysiology of SIRS is explained by the excessive elevation of inflammatory cytokines, which induce the activation and migration of inflammatory cells, which in turn damage the endothelium and cause increased vascular permeability. In past reports ^(20), (21), (22)^, patients with sepsis tended to have higher TNF-α and IL-6 levels. In KD patients ^(3), (23)^, the serum levels of IL-6, IL-10, TNF-α, and IFN-γ are also elevated. TNF-α typically remains elevated after IVIG treatment in patients with CAL. Hence, cytokine activity similar to that seen in septic patients may explain the relationship between SIRS and CAL formation in KD. Some studies of other systemic inflammatory states such as acute pancreatitis ^(24)^ or multiple traumas ^(25)^ have reported that SIRS can predict severity and prognosis. We hypothesize that KD with SIRS is more severe and likely to have a poorer outcome. However, this observation still remains hypothetical because we have not measured the cytokines levels.

Some studies have described changes in the vital signs accompanied by systemic inflammation in KD ^(26), (27)^. Kanegaye et al. reported that KD patients developed shock, higher levels of inflammatory biomarkers, and a higher incidence of CAL ^(28)^. These patients reportedly also exhibited impaired systolic and diastolic function on an echocardiogram, suggesting greater systemic inflammation associated with myocardial involvement. In addition to the cardiogenic factor, another report ^(29)^ described a ‘distributive factor’ due to an increase in vascular permeability. These observations are consistent with our results because SIRS also includes abnormalities in the vital signs.

This study has several limitations. First, the vital signs were measured at one point during triage in the emergency department. According to the SIRS criteria, tachycardia is defined as an elevated pulse in the absence of an external stimulus or as an otherwise unexplained, persistent elevation in the HR. Second, emotional distress and behaviors like crying and sleeping encountered in many pediatric patients often pose a challenge to measuring the vital signs accurately. However, we were unable to assess these behavioral states at the triage since the database did not include this information. For example, KD patients are prone to be bad-tempered; if these patients had been excluded, the disease severity of the cohort might have been milder. Further study involving repeated measurement of the vital signs in the emergency department triage and inpatient ward is needed to assess the persistence of SIRS and to remove the influence of some of the confounding factors. Third, the BT of the patients was measured in the axilla although measuring the core temperature is generally recommended. However, measuring the axillary temperature is common in most of the previous studies of the Kobayashi score. Finally, this study used the Kobayashi score despite its low sensitivity for predicting the severity of the disease in patients outside Japan. Nevertheless, we used the score to compare the severity of the two groups since to date there are no validated, universally applicable scoring systems.

In conclusion, our study suggests that SIRS can be a risk factor for CAL development in the acute phase of KD. Further prospective studies are needed to monitor patients’ vital signs continuously after admission with minimal influence of modifying factors. Recognizing SIRS in pediatric patients will enable early identification and treatment of severe KD patients with a concomitant improvement in the coronary outcomes.

## Article Information

### Conflicts of Interest

None

### Acknowledgement

The authors thank all of the staff of the Clinical Research Support Center and the triage nurses in the Emergency Department at the Tokyo Metropolitan Children’s Medical Center, Tokyo, Japan, and Mr. J.R. Valera for his assistance in editing the manuscript.

### Author Contributions

Yutaro Tomobe conceptualized and analyzed the study, drafted the initial manuscript, and approved the final manuscript as submitted.

Osamu Nomura conceptualized and designed the study, coordinated the data collection instruments, reviewed and revised the manuscript, and approved the final manuscript as submitted.

Yoshihiko Morikawa designed the study, supervised the statistical analyses, reviewed and revised the manuscript, and approved the final manuscript as submitted.

Nobuaki Inoue managed data source, reviewed and revised the manuscript, and approved the final manuscript as submitted.

Hiroshi Sakakibara managed data source, reviewed and revised the manuscript, and approved the final manuscript as submitted.

Masaru Miura supervised this study, critically reviewed and revised the manuscript, and approved the final manuscript as submitted.

### Ethical Statement

This study was approved by the ethics board of the Tokyo Metropolitan Children’s Medical Center (ID: H25-24).
